# Supplementary material for: Importance of subsurface water for hydrological response during storms in a post-wildfire bedrock landscape
Source: Nat Commun. 2023 Jun 29;14:3814. doi: 10.1038/s41467-023-39095-z (PMC10310814; doi:10.1038/s41467-023-39095-z)
Supplement: Supplementary file 1 — Supplementary Information [file 41467_2023_39095_MOESM1_ESM.pdf]

Supplementary Information for

Importance of subsurface water to hydrological response during storms in a post-wildfire bedrock landscape

Abra Atwood[1*], Madeline Hille[2,5*], Marin Kristen Clark[2], Francis Rengers[3], Dimitrios Ntarlagiannis[4], Kirk Townsend[2,6], A. Joshua West[1]

[1]Department of Earth Sciences, University of Southern California, Los Angeles, CA, United States
[2]Department of Earth and Environmental Sciences, University of Michigan Ann Arbor, Ann Arbor, MI, United States
[3]U.S. Geological Survey, Landslide Hazards Program, Golden, CO, United States
[4]Department of Earth and Environmental Sciences, Rutgers University, Newark, NJ, United States
[5]Current address: BGC Engineering, Inc., 600 12th St #300, Golden, CO, United States
[6]Current address: Exponent, Inc., 5401 McConnell Avenue, Los Angeles, CA, United States

*Corresponding authors: Abra Atwood, Madeline Hille; these authors contributed equally.
Email: aatwood@usc.edu, mhille@bgcengineering.ca

This PDF file includes:

Supplementary Text S1-S7
Figures S1 to S13
Tables S1 to S5
SI References

Any use of trade, firm, or product names is for descriptive purposes only and does not imply endorsement by the U.S. Government.

Content

Text S1-S2 discusses precipitation data collected over the study period as well as observations of erosion as they relate to U.S. Geological Survey (USGS) debris flow hazard probabilities.
Text S3 discusses D-excess calculations and results and the methodology behind our weighted precipitation isotope values for individual storm events.
Text S4-S5 covers the methodology behind our resistivity field surveys and inversion process, as well as error considerations.
Text S6 discusses the methodology and findings from evapotranspiration (ET) estimates both pre- and post-fire in all three catchments.
Text S7 discusses the differences in soil surface field-saturated hydraulic conductivity data between catchments.

Figure S1 presents boundary conditions within the chosen catchments (i.e., vegetation index and average slope angle).
Figure S2 compares K_{fs} measurements in both catchments with 15-minute rainfall intensities over the study period.
Figure S3 is a complete presentation of all resistivity tomography inversions in Henry, Louise and Thelma from October 2021- May 2022.
Figure S4 includes photos of observed streamflow between catchments.

Figure S5 shows channel cross sections and methodology for streamflow estimates.
Figure S6 is a complete presentation of all resistivity tomography inversions in Henry, Louise, and Thelma from December 2020-June 2021.
Figure S7 are plots of dual isotopes (δ^{18}O versus δD) and δ^{18}O versus deuterium (D)-excess for streamflow and precipitation samples.
Figure S8. Plot comparing rainfall intensity/median field-saturated hydraulic conductivity (K_{fs}) values to the fraction of new water during December 2021 storms.
Figure S9 presents topographic surveys completed along each catchment with measured depth of highly fractured regolith.
Figure S10 presents an example depth of investigation (DOI) limit of the resistivity surveys, modeled at Thelma usng the December 2020 data.

Figure S11 presents daily evapotranspiration (ET) values from ECOSTRESS data over all three catchments during October 2019-2020 prior to the Bobcat Fire and from October 2020-2021 and October 2021-2022.
Figure S12 is a bar plot with annual ET values derived from Figure S11 using the same daily ECOSTRESS data.
Figure S13 presents photo comparisons of erosion within the catchments.

Table S1 presents catchment boundary conditions averaged over catchment area (i.e., vegetation index, slope, and bedrock geology).
Table S2 lists field-saturated hydraulic conductivity values at catchments Henry, Louise, and Thelma.
Table S3 lists the variances and p-values generated from an F test of K_{fs} measurements at catchments Henry, Louise, and Thelma.
Table S4 lists precipitation and streamflow isotopic data collected during storms.
Table S5 lists resistivity survey and inversion model metrics at catchments Henry, Louise, and Thelma.

Supplementary Information Text

Text S1. Precipitation data

We collected rain gauge data from December 2020 through May 2022 from three gauges. All three recorded similar amounts, and thus rain gauge #3 is the only one reported. Rainfall over the course of (December 2020 -June 2021 totaled about 18.78 cm at rain gauge #1, 18.06 cm at rain gauge #2, and 17.06 cm at rain gauge #3: about 25% of the average annual rainfall total for southern California (~70 cm according to PRISM Climate Group, 2021[1]); October 2021-March 2022 was substantially wetter, ~47 cm, but still below average. Five-minute rainfall amounts were recorded by installed rain gauges. Maximum 15-minute rainfall intensities during each recorded storm event did not exceed U.S. Geological Survey (USGS) debris flow intensity thresholds (see Figures S9 and S10). Maximum 15-minute rainfall intensities did exceed field-saturated hydraulic conductivity (K_{fs}) values during all storms that generated streamflow (Figure S2).

Text S2. Erosion observations

Trail camera footage and field observations showed no signs of a debris flow event in any catchment during 2021. However, substantial sediment evacuation and rilling were observed in early February 2021 following the first two rainstorm events of the season. Up to a meter of sediment was removed from the

burned catchments (Louise and Thelma), exposing scoured bedrock. Debris dams and splash were also observed in early February. Minor sediment movement was observed in the channel of the unburnt catchment (Henry), but no significant patterns were recorded on the hillslopes, which are densely vegetated.

In 2022, during the three December storms, Louise experienced substantial significant streamflow events (Figure S4) that resulted in >2 meters of sediment evacuation in the channel. However, no geomorphic evidence (levees, deposits) of debris flows were present even though much smaller nearby catchments did show minor debris flow deposits. No substantial erosion was observed in Henry over the same time period.

The elevation of each electrode at each survey site was measured after each large storm that resulted in substantial erosion and topographic change in the catchment channels. We used a RTK Septentrio Altus APS3G Global Navigation Satellite System (GNSS) instrument. The accuracy of relative elevation in the same GPS survey is on the order of millimeters[2]. We observed minor-scale surface erosion changes in Henry and Thelma in early February 2021, and a general smoothing of the hillslope surface in Louise with about 0.5-m change in elevation. After the14 December 2021 rainstorm and debris flow event in Louise, substantial topographic change was evident in the GPS survey in the channel. By February 2022, the steep channel walls had relaxed, and the hillslopes and channel returned to a smooth V-shape.

Text S3. D-excess and amount weighted precipitation isotope average values for storms

The stable isotope ratios of water (D/H and $^{18}O/^{16}O$ of water provide information about water sources, offering a tool to trace surface-subsurface connectivity and changes in the source of streamflow over time[3]. Deuterium excess (Dxs) (defined as Dxs=δD−8×δ^{18}O, in ‰) represents the offset from the meteoric water line, and variations are controlled by the kinetics of evaporation. Dxs is especially helpful when variations in δ^{18}O isotopes are small. Plots of Dxs versus δ^{18}O (Figure S7) support the δ^{18}O results, where 10 March, 25 October, and 14 December storms show overlap between streamflow and precipitation, whereas the 15 March and 23 and 24 December storm streamflow show offset from precipitation.

Average amount-weighted precipitation isotope values for each storm were calculated to create end members for each storm event for δ^{18}O and Dxs. Precipitation amounts were binned by 30-minute intervals, and 30-minute average isotope and Dxs values were calculated using a 7-minute offset relative to collection time to reflect an average rainfall time for samples, rather than the time of collection. Average amount-weighted values for precipitation isotopes for each storm ($\delta storm$) were then calculated using the following equation[4]:

$$\delta storm = \sum_{storm\ end}^{storm\ end} \delta 30min \times \frac{P_{30min}}{P_{storm\ total}}$$

where $\delta 30min$ were the average isotopic values every 30 minutes, starting from initial rainfall, P_{30min} is the binned 30-minute amount of rain, and $P_{storm\ total}$ is the total amount of rain that fell during the storm event.

Text S4. Electrical resistivity imaging (ERI) data processing and inversion parameters

Across the SGM study catchments, geologic properties remain relatively uniform both laterally within each catchment and with depth, and starting conditions are highly resistive. Therefore, ERI is an ideal method for observing shallow subsurface water storage[5,6] that is typically too deep to monitor with soil moisture

sensors. Further, the short-term deployment of the resistivity array enables surveys within the steep and unstable terrain of our study catchments, which would otherwise be challenging to instrument for soil moisture.

Resistivity survey data were downloaded from the instrument and filtered for outlier data points in Prosys II software from Iris Instruments. We removed negative apparent resistivity values and erroneously high resistivity data points in both normal and reciprocal surveys such that >90% of the data was still used in each inversion model. Topography was added during the inversion process.

Inversion models were produced in ResIPy open-source software[7] with a regularized inversion with linear filtering, a model-to-data root mean square error (RMSE) tolerance value between 1 and 1.5 and weight parameters *a* and *b*, which were derived from a power-law error model where absolute reciprocal error is a function of resistance, which gives a better representation of the subsurface and a well-constrained model of resistivity[8–10]. A fine-scale triangular mesh was used to accommodate topography. Time-lapse models were also produced in ResIPy with reciprocal measurements using the difference inversion approach[11].

Depth of investigation (DOI) is the maximum depth at which the resistivity modeled data are controlled by the collected data; below the DOI, the modeled data are not controlled by the collected data and are considered inversion constructs[12]. We estimated the DOI for our surveys to ensure that the observed changes are representative of the data, and not inversion artifacts. Figure S10 shows the DOI for the Thelma December 2020 data, as estimated by the method proposed by Oldenburg and Li (1999)[12] suggesting that all observed changes are well within the modeled area controlled by the data. The rest of the surveys also show similar DOIs.

Text S5. Electrical resistivity imaging error considerations.

A highly resistive environment like the soils, regolith, and fractured gneissic bedrock of the San Gabriel Mountains lends itself to tracking moisture contrasts in resistivity[13] but presents similarly high contact resistance between the electrodes and the shallow ground surface. Low contact resistance is desirable for strong electrical conductance and a consistent electrical current in the subsurface; high contact resistances can lead to erroneous data points and collection error[14]. While there is not a set threshold for good contact resistance, the lower the value, the better for data quality. The actual threshold is also defined by the survey objective and environmental conditions. In the driest months of our study period (early December and March onward), we encountered contact resistances regularly ranging between 100 and 500 kohms. To reduce contact resistance between the electrode probes and the dry, gravelly near surface, we used a combination of bentonite clay cat litter and water packed around each electrode, which reduced most contact resistances to <60 kohms (Table S5). We used ~50 kohms as our acceptable threshold where practical, primarily because of time constraints and efficiency. The highest contact resistances consistently registered at electrodes located in materials with extensive air space (e.g., gravelly soils).

Reciprocal measurements are the standard quantification of data collection error for ERI[8,9]. Separate reciprocal surveys with ~⅓ data density were paired with normal surveys and individual power-law error models built for each weighted inversion. Absolute reciprocal error is modeled as a function of resistivity in 20-point bins of increasing resistance. R-squared values of the error models range between 0.767 and 0.972 (Table S5), and the output is used to calculate inversion weight parameters *a* and *b*.

Environmental factors such as temperature and salinity can strongly affect resistivity[15], especially when calculating soil water content. We collected a limited set of temperature data for the Thelma catchment for an ~ 3-month period (12/2020 – 2/2021) that show the dominant air temperature changes, averaged daily, to be ~ ±3 ^{O}C, with 2 spikes ~ ±7 ^{O}C during this period. Because the SGM is a Mediterranean environment, the largest temperature fluctuations are expected during the winter months. Considering an estimated 2% change for every degree ^{O}C[16], we expect a maximum influence of ~14% on the resistivity data, limited to the top 1 m. This is a small fraction compared to the changes observed, and is also representative of diurnal temperature swings, whereas we collected resistivity surveys during daylight hours. Temperature is not considered to be the main driver of the observed changes, although it may have partially contributed.

Here, we do not attempt to calculate soil water content or water volume because we do not have the necessary soil temperature or porosity data to do so, and the uncertainties of this approach are large in a locally variable environment like the shallow subsurface within the San Gabriel Mountains[17–19]. Instead, we simply interpret changes to resistivity as relative addition or removal of water to the subsurface under the assumption that very little water was present in the early Fall.

Text S6. Evapotranspiration (ET) estimates

The 70-m resolution ECOSTRESS ET data derived from the PT-JPL algorithm (ECO3ETPTJPL) were downloaded from 1 October 2019 to 1 October 2022 using the Land Processes Distributed Active Archive Center (LP DAAC) Application for Extracting and Exploring Analysis Ready Samples (AppEEARS)[20]. ECOSTRESS data have been used in past postfire paired catchment studies to quantify ET in southern California[21]. ECOSTRESS rasters were processed in Python using rasterstats zonal_stats() function to calculate the median ET values for each catchment. ET rasters were screened for quality assurance flags as well as sites where ET was three standard deviations greater than the average. In total, 114 rasters were used in this study. Daily ET measurements in W/m^2 are presented in Figure S11. Annual ET measurements are presented in Figure S12.

ECOSTRESS ET shows similar annual ET values in all three catchments during the water year before the Bobcat Fire. In WY1, ET was reduced in the unburned site relative to the year before, likely because WY1 was drier. Greater reductions are seen in both burned catchments in WY1. WY1 annual ET is ~2x higher in the unburned catchment than the burned catchments, but in WY2 all catchments show similar ET amounts (Figure S12).

The difference in ET between the catchments corroborate the large increase in streamflow and subsurface storage. Given that these are all ephemeral catchments, ET likely dominates the water budgets with limited water exiting as streamflow. Therefore, if ET is only half of what it would normally be, the amount available to go into subsurface storage and streamflow would increase by substantially more proportionally because they make up much smaller fractions of the overall budget. Although a complete water budget calculation is unavailable due to lack of streamflow measurements, such a drop in ET as seen from this data could lead to greater streamflow as well as increased subsurface storage. Other post-fire studies in southern California have found ET made up as much as 90% of the water budget in the unburned catchment (994 mm/year ET; 102 mm/year runoff) and also found larger changes in storage in the burned catchment and higher streamflow[21].

Text S7. Soil surface field-saturated hydraulic conductivity

Median K_{fs} values of soil surface field-saturated hydraulic conductivity are similar between burned and unburned catchments, but the distributions of K_{fs} values show greater variability in the burned catchments, consistent with previous studies[22–26]. Variability of K_{fs} in the burned catchments likely reflects the creation of a spatially variable hydrophobic layer (low K_{fs}) during combustion, which locally decreases K_{fs}, as well as the removal of debris from the soil surface during the fire, which locally increases K_{fs} [25,27].

The combined post-fire effect is expected to result in point locations with very high hydrophobicity, as well as some locations with very low hydrophobicity where vegetation debris and duff have been removed. By contrast, low K_{fs} values in the unburned catchment likely reflect locations where vegetation debris and duff prevent infiltration[28]. Rainfall intensities did not exceed measured K_{fs} during all storms, yet we observed the isotopic signature of new water; in these instances, overland flow may be driven by saturation of the upper 5-10 cm of soil during successive storms rather than rainfall exceeding infiltration rates (Figure S2)[29].

Figures

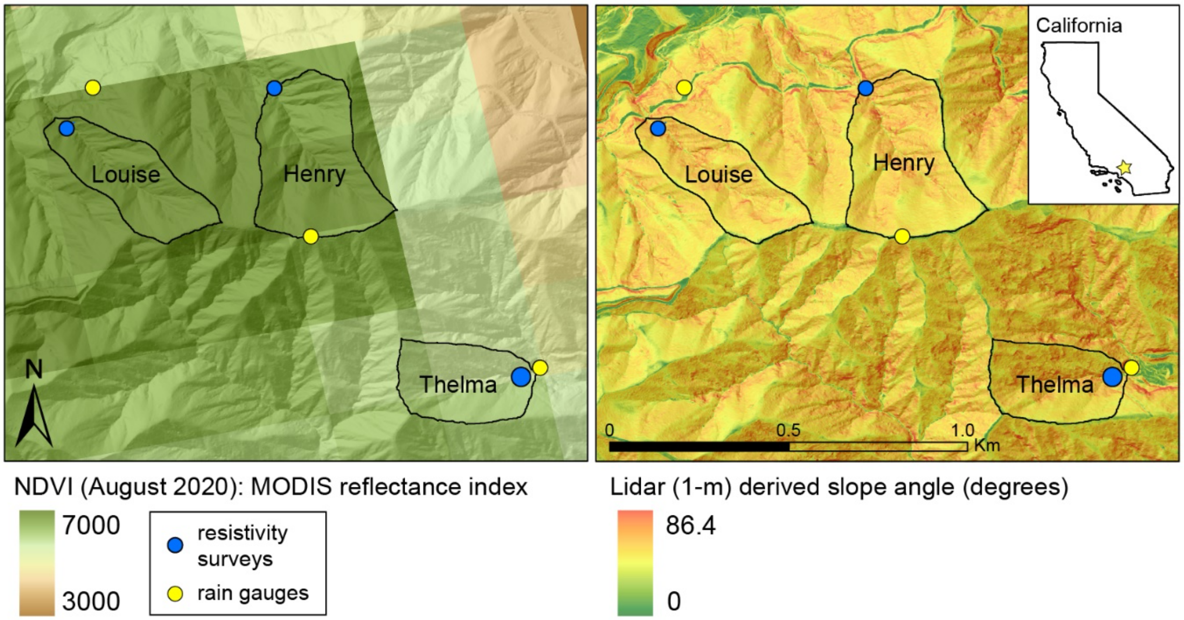

Figure S1. Catchment boundary conditions including normalized difference vegetation index (NDVI), and slope angle. Louise and Henry were more heavily vegetated pre-fire (August 2020 NDVI: ~7000) with chaparral-type deciduous shrubbery, manzanita, and oak trees (MODIS reflectance data). Thelma was less densely vegetated with a pre-fire NDVI index of ~6300 and fewer large oak trees. Lidar-derived slope is based on USGS 3DEP services provided by the OpenTopography Facility with support from the National Science Foundation under NSF Award Numbers 1833703, 1833643, and 1833632. Lidar dataset Name: USGS LPC CA Los Angeles 2016 LAS 2018.

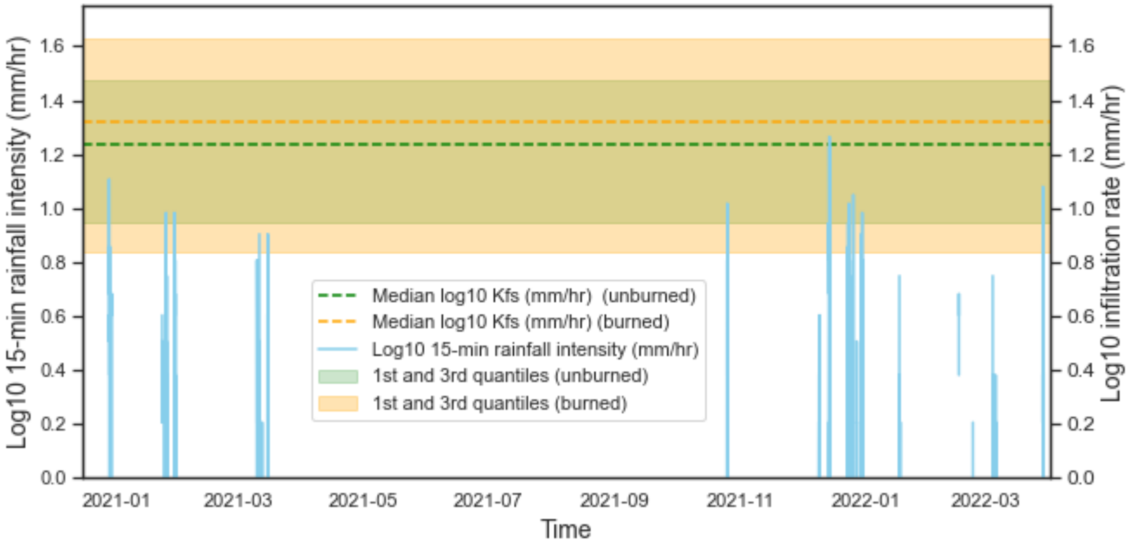

Figure S2. Comparison of field-saturated hydraulic conductivity (K_{fs}) in catchments Louise and Henry with rainfall rates over the study period.

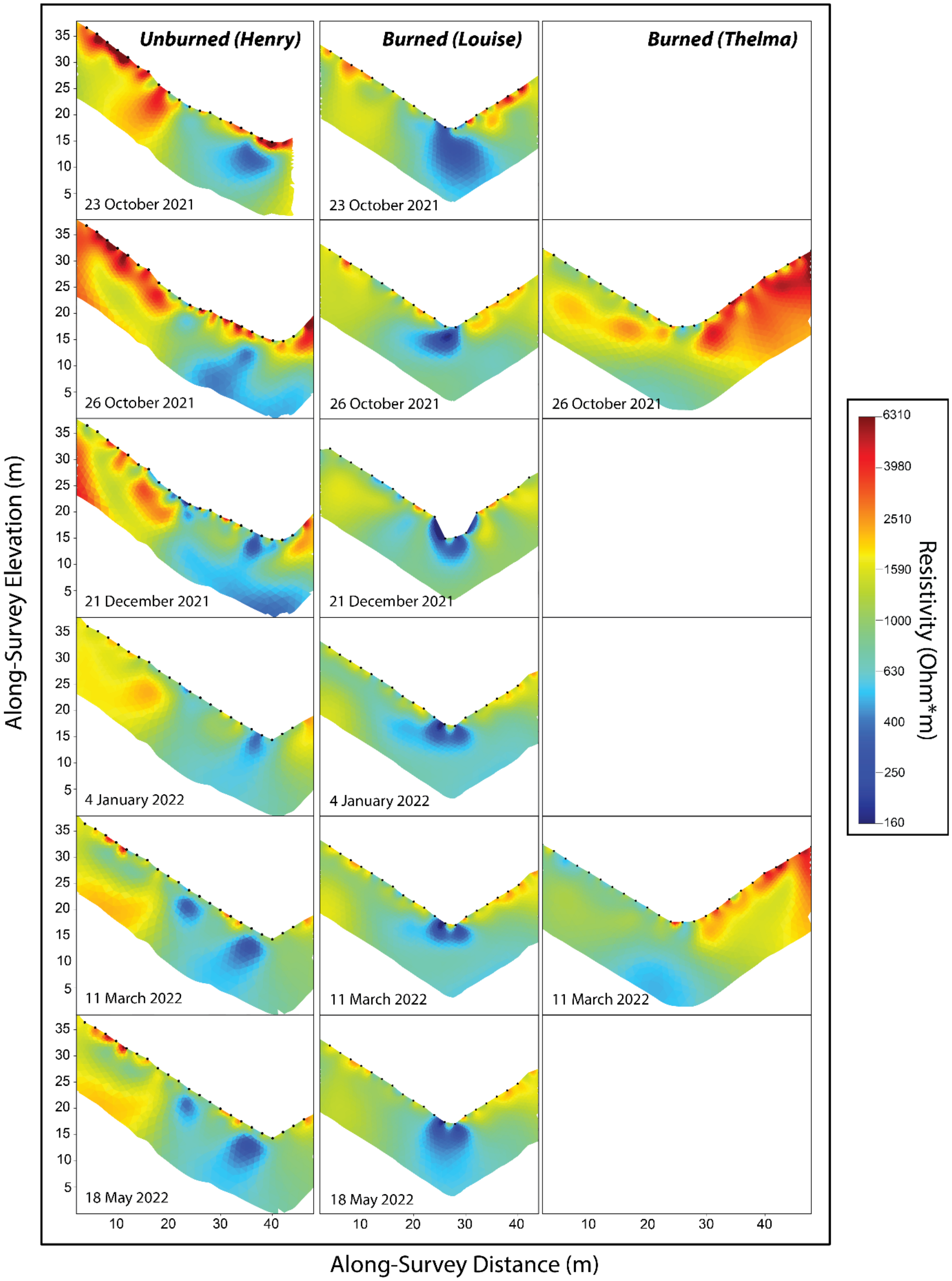

Figure S3. Electrical resistivity inversion models at Henry, Louise, and Thelma between October 2021 and May 2022.

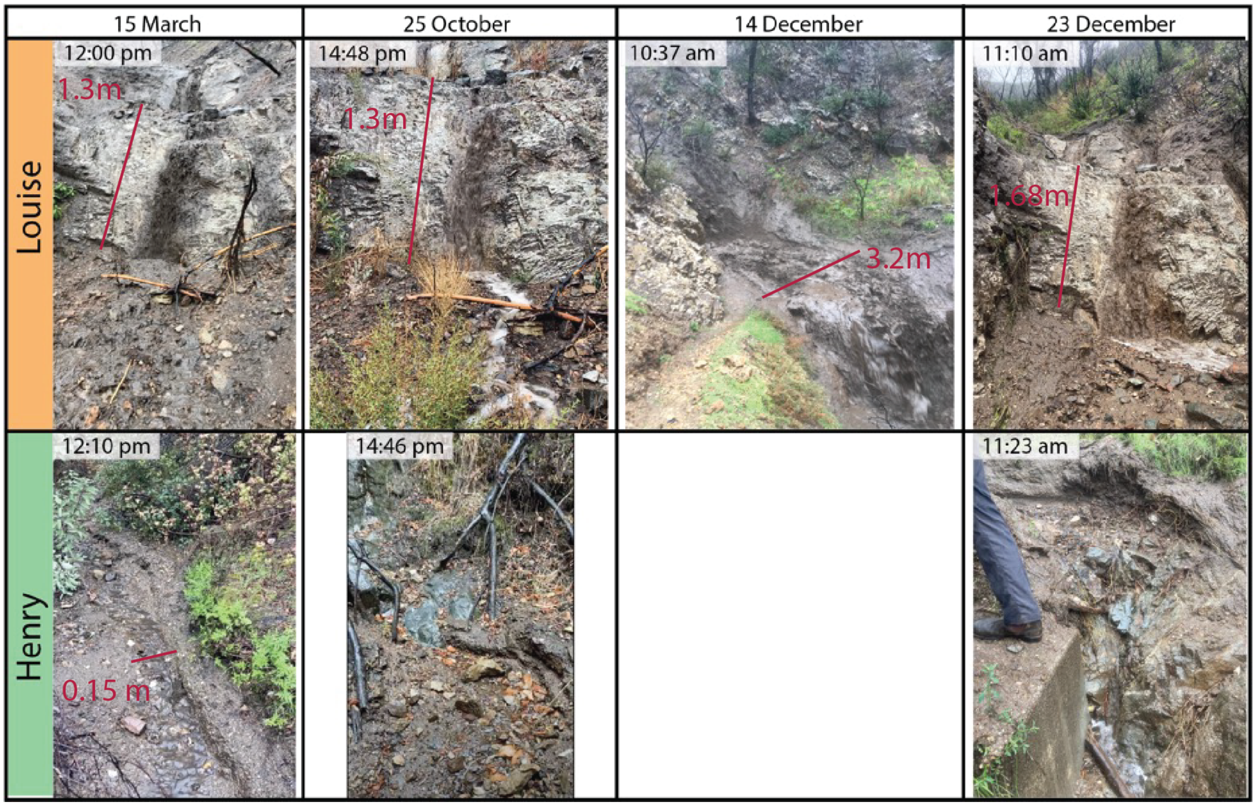

Figure S4. Streamflow comparisons between burned (Louise) and unburned (Henry) catchments during different storm events. Camera broke during the 14 December storm so photograph is not available. Photos courtesy of A. Atwood.

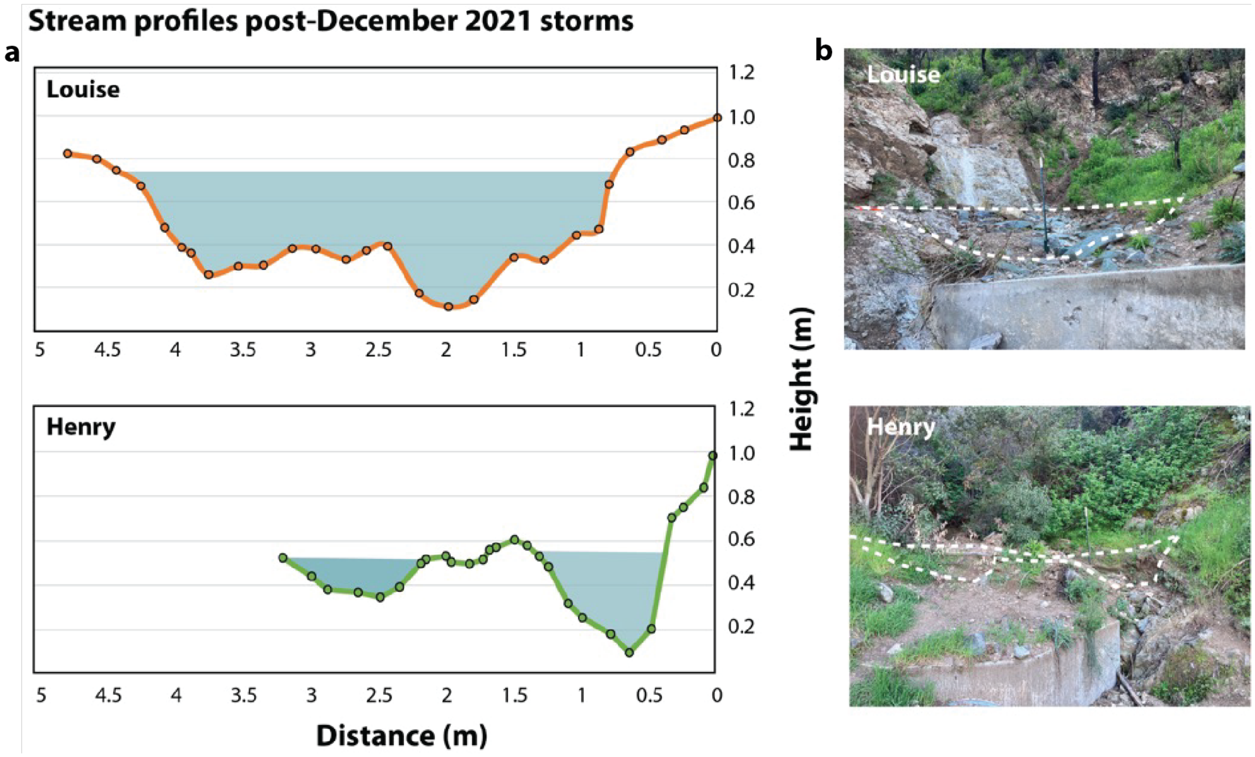

Figure S5. (a) Stream profiles (orange and green lines) using an RTK Septentrio GPS instrument for maximum streamflow estimation for December 2021 storms. Blue indicates estimated cross-sectional area of streams. (**b)** Photos of location where stream profiles were done. White dashed lines indicate stream profiles in (**a**)**.** Note that stream profiles were taken directly above a hydraulic jump at both locations. Photos courtesy of A. Atwood.

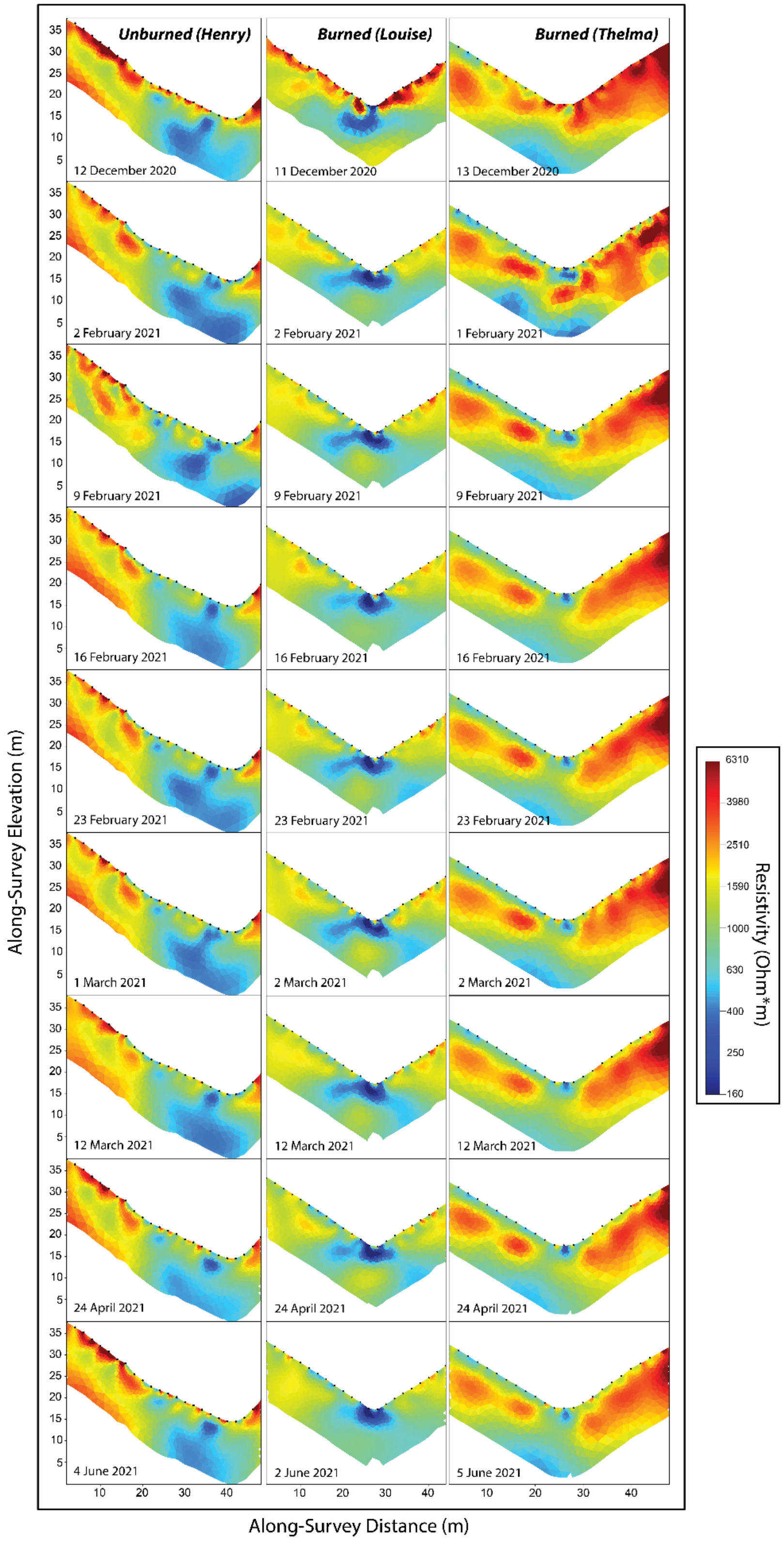

Figure S6. Electrical resistivity inversion models at Henry, Louise, and Thelma between December 2020 and June 2021.

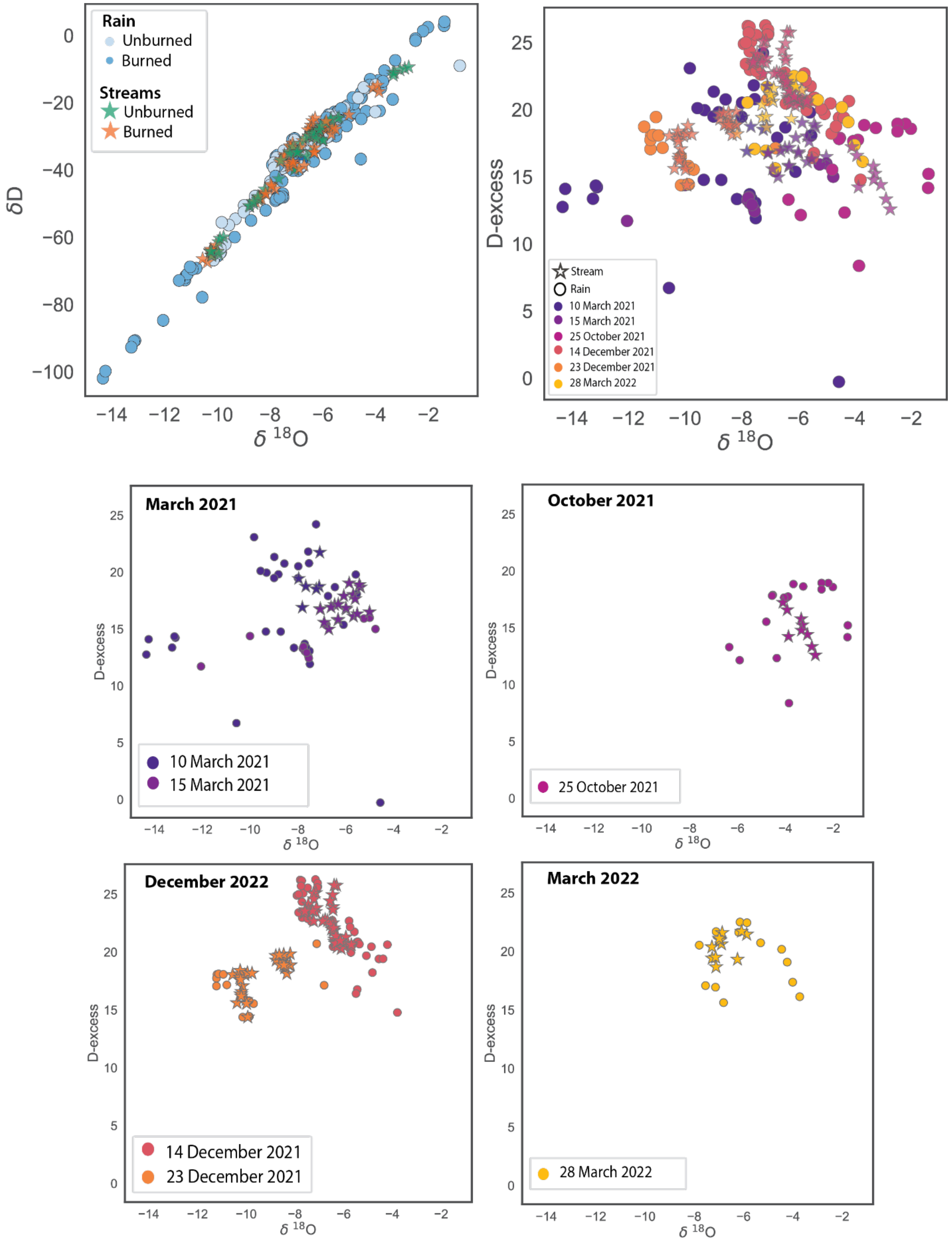

Figure S7. Dual isotope plot (δD and δ^{18}O) and deuterium (D)-excess versus δ^{18}O. D-excess versus δ^{18}O monthly plots show rainfall isotope values weighted by concurrent precipitation amount.

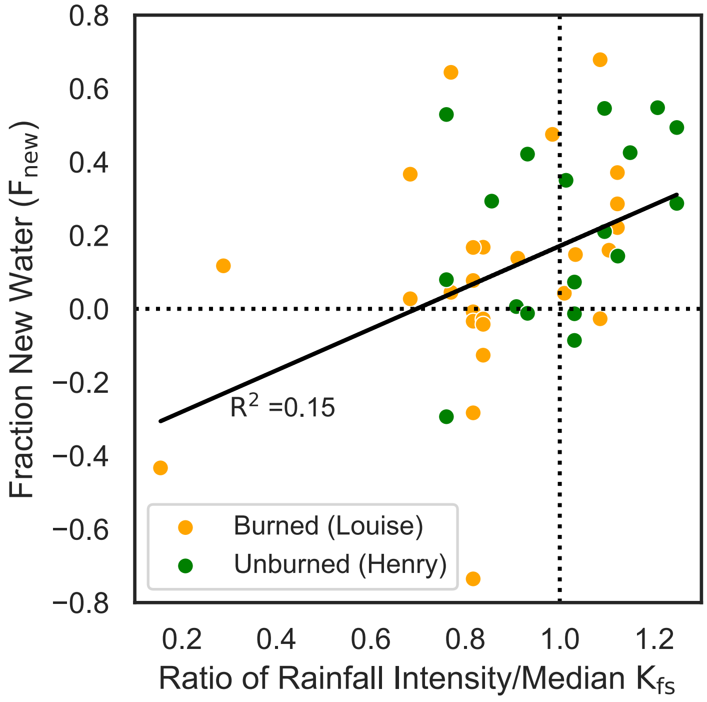

Figure S8. Plot comparing rainfall intensity/median values of field-saturated hydraulic conductivity (K_{fs}) to the fraction of new water during December 2021 storms. New water fraction roughly correlates with rainfall intensities that exceed median K_{fs} values in each catchment, although more scatter in F_{new} is observed in the burned catchment when rainfall intensities do not exceed median K_{fs}, which is consistent with more variable post-fire K_{fs}.

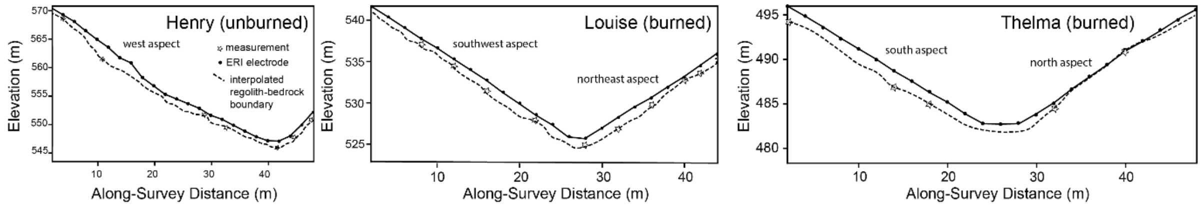

Figure S9. Topographic surveys completed along each catchment, showing ERI survey electrodes (dots) and measured depth-to-intact bedrock (stars). Measurements were done by pounding rebar stakes into the surface. The point of resistance was measured and is interpreted here as a plausible regolith boundary where bedrock fracture density changes.

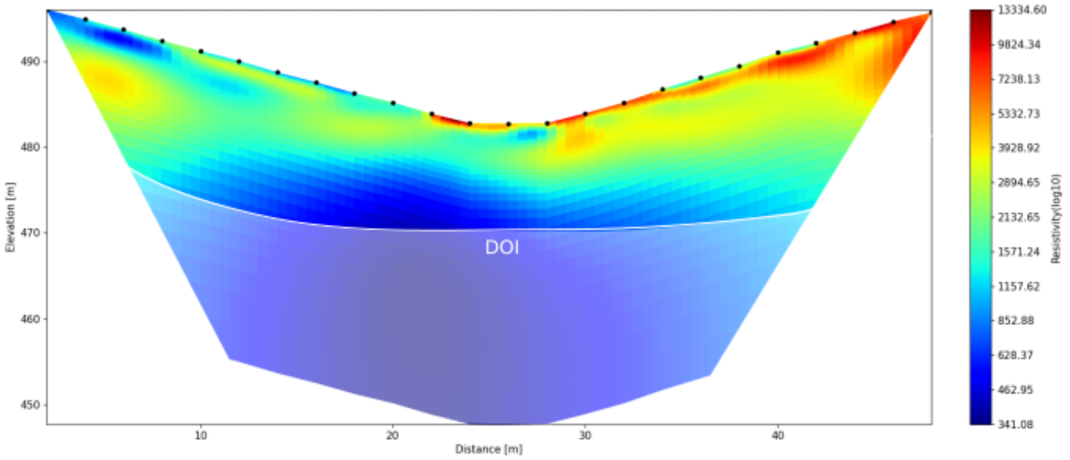

Figure S10. Depth of investigation (DOI) for the 13 December 2020 survey at the Thelma catchment.

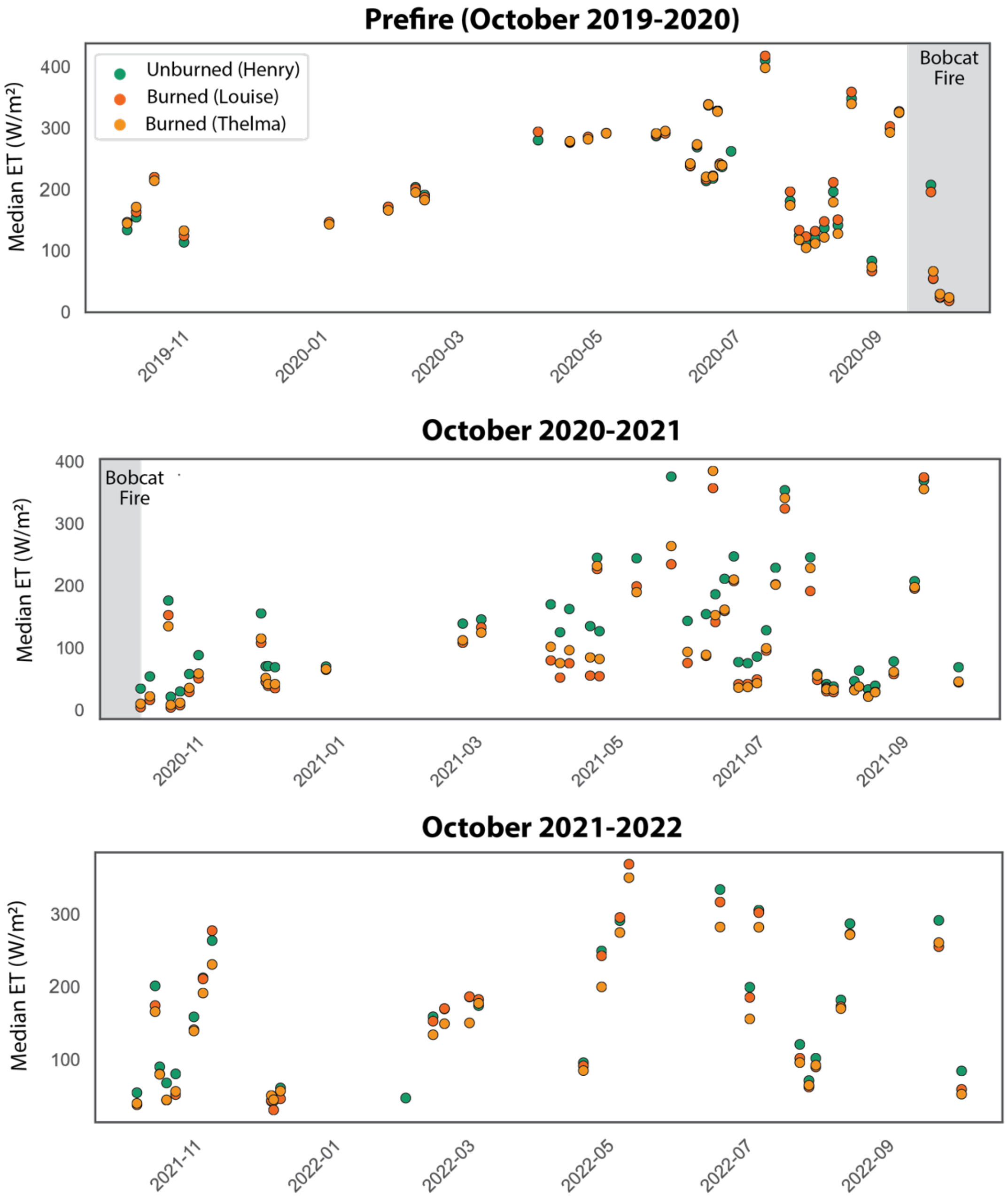

Figure S11. Daily median ET values from ECOSTRESS data[20] colored by catchment. All catchments have similar pre-fire ET, whereas from Oct 2020-2021 (postfire) the unburned catchment shows higher ET during most days captured by ECOSTRESS. This increase is less pronounced from Oct 2021-2022, when vegetation recovery in the burned catchments increased.

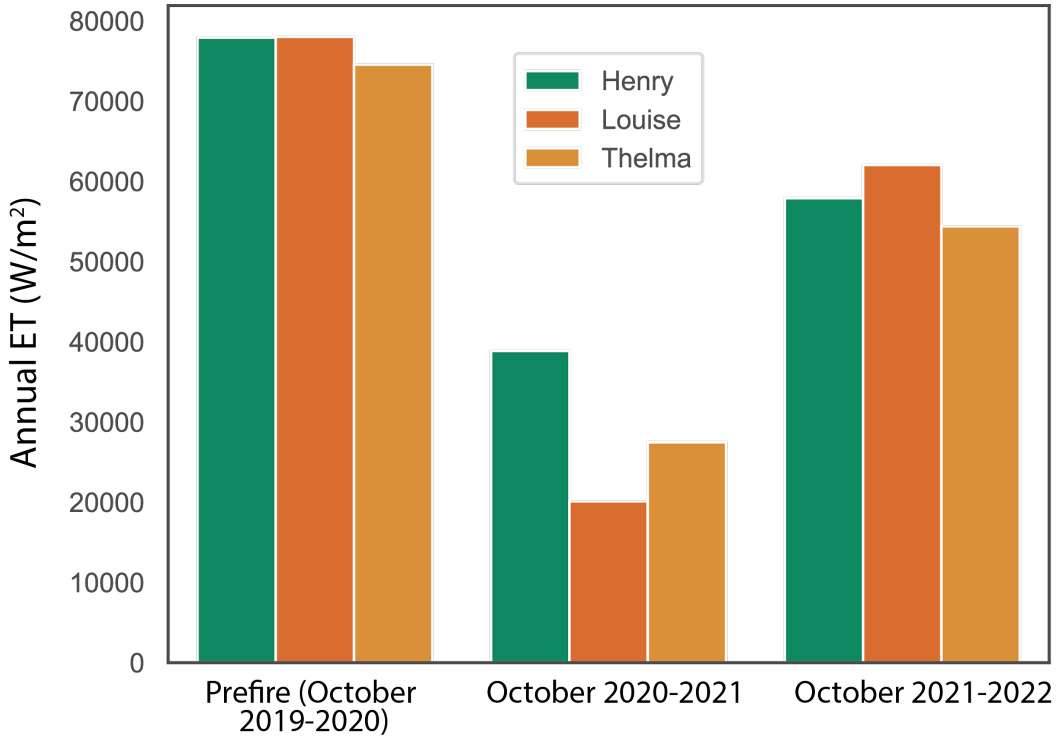

Figure S12. Annual ET over each catchment during the pre-fire (October 2019-2020), Oct 2020-2021 (postfire), and Oct 2021-2022.

Photo comparison of dry-ravel “loaded” catchment channels in December 2020 and erosion in WY1 and WY2

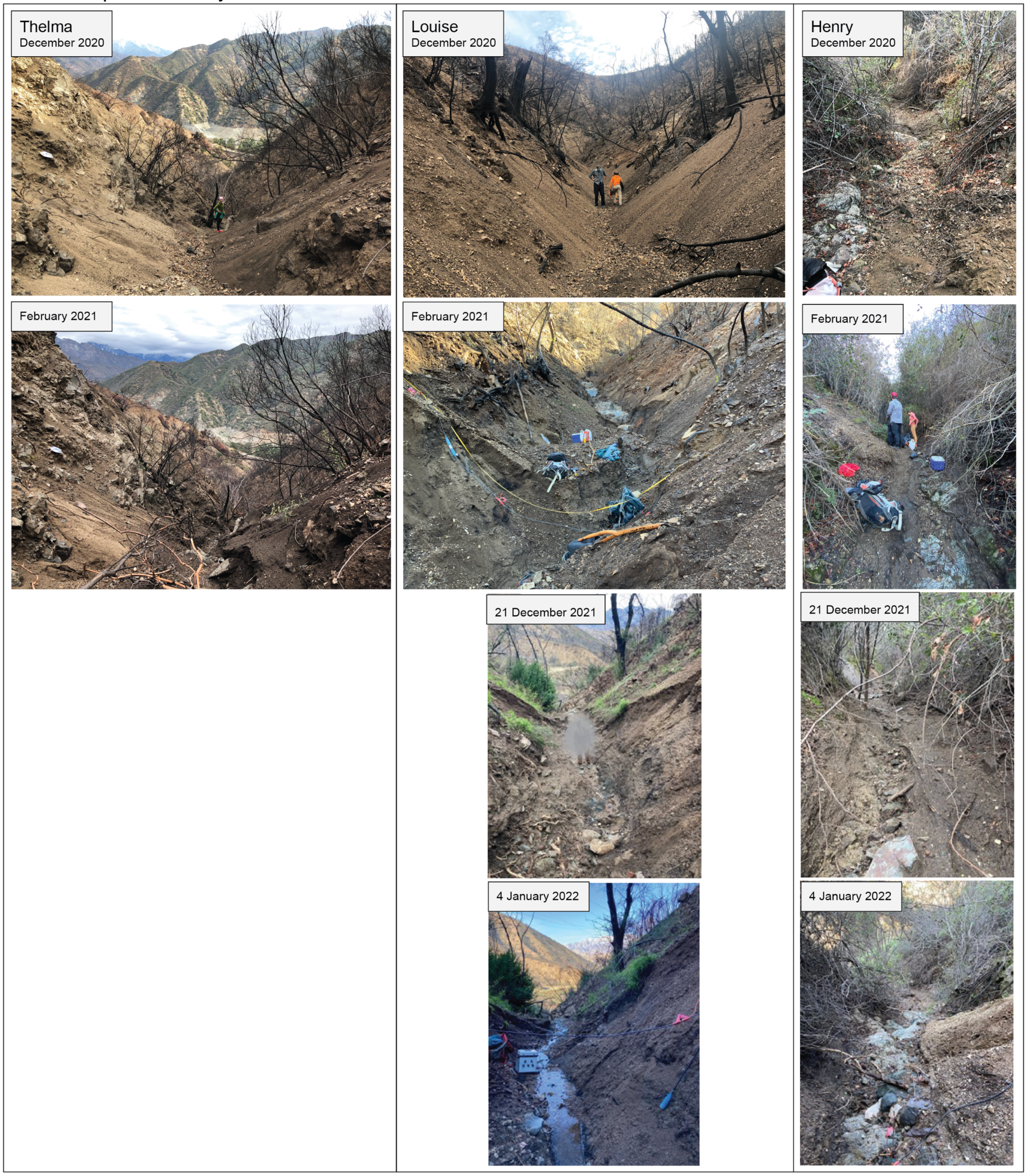

Figure S13. Photo comparison of catchments before and after storms in WY1 and WY2. Photo credits: M. Hille and A. Atwood.

Tables

Table S1. Catchment boundary conditions, averaged over catchment area. Lidar-derived metrics are based on USGS 3DEP services provided by the OpenTopography Facility with support from the National Science Foundation under NSF Award Numbers 1833703, 1833643, and 1833632. Lidar dataset Name: USGS LPC CA Los Angeles 2016 LAS 2018.

Catchment	Percent of catchment unburned or burned at low severity (%)	Percent of catchment burned at moderate severity (%)	Percent of catchment burned at high severity (%)	Pre-fire NDVI (MODIS reflectance data; August 2020 area-averaged)	Lidar-derived slope (avg; degrees)	Average normalized channel steepness (K_{sn})	Area (km^2)
Thelma	0	63.6	36.4	6304	39.44	29.0	0.0594
Louise	0	27.0	73	7080	39.59	29.8	0.0652
Henry	76.2	18.2	5.6	6973	39.48	33.0	0.1011

Table S2. Field-saturated hydraulic conductivity (K_{fs}) values from catchments Henry, Louise, and Thelma. Zero values indicate no infiltration after 45 minutes.

Sample ID	Catchment	Collection Date	Kfs (mm/hr)
LMD1	Louise	26 February 2021	0
LMD3	Louise	26 February 2021	0
LMD5	Louise	26 February 2021	2.98
LMD6	Louise	26 February 2021	11.96
LMD7	Louise	26 February 2021	8.33
LMD8	Louise	26 February 2021	5.74
LMD9	Louise	26 February 2021	21.86
LMD10	Louise	26 February 2021	20.97
LMD11	Louise	31 August 2021	0.97
LMD12	Louise	31 August 2021	106.49
LMD13	Louise	31 August 2021	90.87
LMD14	Louise	31 August 2021	34.6

LMD15	Louise	31 August 2021	17.93
LMD16	Louise	31 August 2021	48.62
LMD17	Louise	31 August 2021	40.49
LMD18	Louise	31 August 2021	45.21
LMD19	Louise	31 August 2021	20.37
LMD20	Louise	31 August 2021	21.51
LMD21	Louise	31 August 2021	62.96
HMD1	Henry	31 August 2021	2.29
HMD2	Henry	31 August 2021	14.85
HMD4	Henry	31 August 2021	24.54
HMD5	Henry	31 August 2021	43.6
HMD6	Henry	31 August 2021	8.35
HMD7	Henry	31 August 2021	10.64
HMD9	Henry	31 August 2021	248.92
HMD10	Henry	31 August 2021	0
HMD11	Henry	31 August 2021	31.75
HMD12	Henry	31 August 2021	0
HMD13	Henry	31 August 2021	15.43
HMD14	Henry	31 August 2021	33.64
HMD15	Henry	31 August 2021	20.98
HMD16	Henry	31 August 2021	19.16
HMD17	Henry	31 August 2021	47.84
HMD18	Henry	31 August 2021	14.72
HMD20	Henry	31 August 2021	0

HMD21	Henry	31 August 2021	20.81
MD1	Thelma	25 February 2021	11.45
MD2	Thelma	25 February 2021	39.84
MD3	Thelma	25 February 2021	28.37
MD4	Thelma	25 February 2021	11.15
MD5	Thelma	25 February 2021	12.66
MD6	Thelma	25 February 2021	15.07
MD7	Thelma	25 February 2021	49.04
MD8	Thelma	25 February 2021	25.88
MD9	Thelma	25 February 2021	55.61
MD10	Thelma	25 February 2021	11.26
MD11	Thelma	3 April 2021	27.61
MD12	Thelma	3 April 2021	8.3
MD14	Thelma	3 April 2021	101.54
MD15	Thelma	3 April 2021	48.76
MD16	Thelma	3 April 2021	36.18
MD17	Thelma	3 April 2021	17.5
MD18	Thelma	3 April 2021	54.54
MD19	Thelma	3 April 2021	39.04
MD20	Thelma	3 April 2021	96.96
MD21	Thelma	3 April 2021	11.73
E11	Thelma	25 February 2021	17.31
E15	Thelma	25 February 2021	1.68

Table S3. Statistical assessment of variance between field-saturated hydraulic conductivity (K_{fs}) values measured in catchments Henry, Louise and Thelma.

Catchment	Variance	Catchment Comparison	P-value
Thelma	688.3	Louise vs. Henry	0.0026
Louise	871.7	Thelma vs. Henry	0.0083
Henry	202.0	Louise vs. Thelma	0.2937

Table S4. Isotopic data collected during storm events from precipitation and streamflow samples, concurrent rainfall amounts, and storm identification. “SD” shows standard deviation.

**Sample Name**	**Date**	**Time**	**Sample Type**	**Location**	**Storm**	**Rain (cm)**	**δ^{18}O**	**SD δ^{18}O**	**δD**	**SD δD**
HR-1	3/10/21	3:21	Rain	Henry	storm 1	0.22	-7.536	0.023	-39.499	0.099
HR-2	3/10/21	3:48	Rain	Henry	storm 1	0.12	-8.815	0.018	-50.709	0.031
HR-3	3/10/21	4:17	Rain	Henry	storm 1	0.1	-8.566	0.024	-47.762	0.104
HR-4	3/10/21	5:06	Rain	Henry	storm 1	0.1	-7.986	0.032	-43.364	0.017
HR-5	3/10/21	5:36	Rain	Henry	storm 1	0.2	-8.986	0.013	-50.543	0.005
HR-6	3/10/21	6:03	Rain	Henry	storm 1	0.08	-9.312	0.006	-54.532	0.029
HR-7	3/10/21	6:34	Rain	Henry	storm 1	0.02	-9.561	0.033	-56.372	0.024
HR-8	3/10/21	7:06	Rain	Henry	storm 1	0.02	-8.996	0.009	-52.476	0.029
HR-9	3/10/21	11:20	Rain	Henry	storm 1	0.02	-8.000	0.025	-44.524	0.168
HR-10	3/10/21	14:03	Rain	Henry	storm 1	0.02	-6.096	0.044	-33.389	0.100
HR-11	3/10/21	14:30	Rain	Henry	storm 1	0.16	-5.539	0.036	-26.227	0.132
HR-12	3/10/21	14:53	Rain	Henry	storm 1	0.02	-5.584	0.016	-24.875	0.087

HR-13	3/10/21	17:47	Rain	Henry	storm 1	0.12	-7.242	0.012	-33.723	0.079
HR-14	3/10/21	23:10	Rain	Henry	storm 1	0.22	-7.570	0.079	-38.743	0.320
HR-15	3/10/21	23:48	Rain	Henry	storm 1	0.14	-9.832	0.044	-55.569	0.082
LR-1	3/10/21	3:06	Rain	Louise	storm 1	0.22	-14.333	0.036	-101.908	0.149
LR-2	3/10/21	3:36	Rain	Louise	storm 1	0.2	-4.559	0.009	-36.740	0.192
LR-3	3/10/21	4:03	Rain	Louise	storm 1	0.1	-13.116	0.017	-90.697	0.218
LR-4	3/10/21	4:33	Rain	Louise	storm 1	0.06	-7.628	0.008	-48.110	0.138
LR-5	3/10/21	5:24	Rain	Louise	storm 1	0.22	-7.514	0.018	-47.047	0.006
LR-6	3/10/21	5:50	Rain	Louise	storm 1	0.1	-13.159	0.016	-90.924	0.131
LR-7	3/10/21	6:15	Rain	Louise	storm 1	0.04	-9.340	0.023	-59.943	0.082
LR-8	3/10/21	6:48	Rain	Louise	storm 1	0.2	-7.659	0.028	-47.880	0.045
LR-9	3/10/21	11:38	Rain	Louise	storm 1	0.02	-8.165	0.009	-51.975	0.008
LR-10	3/10/21	14:17	Rain	Louise	storm 1	0.06	-7.718	0.019	-48.044	0.033
LR-11	3/10/21	14:42	Rain	Louise	storm 1	0.12	-14.239	0.005	-99.808	0.141
LR-12	3/10/21	17:28	Rain	Louise	storm 1	0.12	-8.719	0.009	-54.978	0.124
LR-13	3/10/21	22:56	Rain	Louise	storm 1	0.16	-10.570	0.020	-77.838	0.368
LR-14	3/10/21	23:31	Rain	Louise	storm 1	0.16	-7.501	0.023	-48.086	0.033
HS-1	3/10/21	23:14	Stream	Henry	storm 1		-7.085	0.020	-34.937	0.121
HS-2	3/10/21	23:48	Stream	Henry	storm 1		-7.671	0.010	-42.617	0.033
LS-1	3/10/21	3:06	Stream	Louise	storm 1		-7.111	0.030	-38.149	0.072
LS-2	3/10/21	5:24	Stream	Louise	storm 1		-7.969	0.010	-44.313	0.050
LS-3	3/10/21	22:52	Stream	Louise	storm 1		-7.232	0.027	-39.332	0.076
LS-4	3/10/21	23:31	Stream	Louise	storm 1		-7.816	0.062	-45.630	0.217

LR-15	3/11/21	0:03	Rain	Louise	storm 1	0.06	-13.257	0.020	-92.688	0.080
HR-16	3/11/21	0:07	Rain	Henry	storm 1	0.08	-6.454	0.024	-32.949	0.088
HR-17	3/11/21	0:22	Rain	Henry	storm 1	0.06	-6.743	0.049	-36.036	0.118
LR-23	3/15/21	7:45	Rain	Louise	storm 2	0.04	-12.052	0.007	-84.700	0.166
LR-24	3/15/21	8:15	Rain	Louise	storm 2	0.04	-10.003	0.008	-65.637	0.086
LR-25	3/15/21	8:35	Rain	Louise	storm 2	0.06	-7.745	0.022	-48.454	0.104
LR-26	3/15/21	8:45	Rain	Louise	storm 2	0.04	-5.003	0.012	-24.028	0.122
LR-27	3/15/21	9:00	Rain	Louise	storm 2	0.06	-4.759	0.079	-23.067	0.289
LR-28	3/15/21	9:20	Rain	Louise	storm 2	0.08	-5.242	0.029	-26.014	0.061
LR-16	3/15/21	11:10	Rain	Louise	storm 2	0.04	-7.798	0.013	-49.094	0.068
LR-17	3/15/21	11:26	Rain	Louise	storm 2	0.12	-7.705	0.025	-48.599	0.149
LR-18	3/15/21	11:41	Rain	Louise	storm 2	0.1	-7.767	0.012	-48.702	0.040
LR-19	3/15/21	11:51	Rain	Louise	storm 2	0.1	-7.565	0.006	-47.916	0.088
LR-20	3/15/21	12:00	Rain	Louise	storm 2	0.12	-7.542	0.017	-47.905	0.090
LR-21	3/15/21	16:10	Rain	Louise	storm 2	0.08	-7.632	0.028	-48.139	0.110
LR-22	3/15/21	16:46	Rain	Louise	storm 2	0.2	-6.816	0.014	-39.112	0.101
HS-3	3/15/21	11:57	Stream	Henry	storm 2		-5.690	0.022	-27.485	0.122
HS-4	3/15/21	12:30	Stream	Henry	storm 2		-5.983	0.015	-31.125	0.025
HS-5	3/15/21	12:42	Stream	Henry	storm 2		-6.083	0.034	-30.749	0.188
HS-6	3/15/21	12:50	Stream	Henry	storm 2		-6.467	0.006	-34.598	0.113
HS-7	3/15/21	13:17	Stream	Henry	storm 2		-7.061	0.012	-39.700	0.105
HS-8	3/15/21	13:50	Stream	Henry	storm 2		-7.070	0.016	-39.814	0.095
HS-9	3/15/21	16:18	Stream	Henry	storm 2		-5.994	0.022	-31.105	0.027

HS-10	3/15/21	16:40	Stream	Henry	storm 2		-5.384	0.016	-24.388	0.057
HS-11	3/15/21	17:00	Stream	Henry	storm 2		-5.435	0.009	-24.585	0.049
HS-12	3/15/21	17:20	Stream	Henry	storm 2		-6.617	0.030	-36.013	0.062
LS-5	3/15/21	11:47	Stream	Louise	storm 2		-5.859	0.083	-27.833	0.262
LS-6	3/15/21	12:02	Stream	Louise	storm 2		-5.679	0.022	-29.318	0.064
LS-7	3/15/21	12:18	Stream	Louise	storm 2		-6.316	0.023	-33.354	0.029
LS-8	3/15/21	12:38	Stream	Louise	storm 2		-6.329	0.020	-34.844	0.131
LS-9	3/15/21	13:00	Stream	Louise	storm 2		-6.710	0.003	-38.694	0.081
LS-10	3/15/21	13:30	Stream	Louise	storm 2		-6.902	0.014	-39.629	0.012
LS-11	3/15/21	16:27	Stream	Louise	storm 2		-5.610	0.029	-27.232	0.081
LS-12	3/15/21	16:50	Stream	Louise	storm 2		-5.008	0.052	-23.564	0.155
LS-13	3/15/21	17:07	Stream	Louise	storm 2		-5.522	0.026	-27.837	0.094
LS-14	3/15/21	17:30	Stream	Louise	storm 2		-6.320	0.002	-34.716	0.052
OLR-1	10/25/21	8:30	Rain	Louise	storm 3	0.02	-1.405	0.019	2.936	0.132
OLR-2	10/25/21	9:30	Rain	Louise	storm 3	0.08	-1.390	0.019	4.100	0.108
OLR-3	10/25/21	10:30	Rain	Louise	storm 3	0.12	-2.015	0.039	2.460	0.062
OLR-4	10/25/21	11:30	Rain	Louise	storm 3	0.16	-2.204	0.020	1.313	0.037
OLR-5	10/25/21	12:10	Rain	Louise	storm 3	0.28	-2.487	0.020	-0.945	0.085
OLR-6	10/25/21	13:00	Rain	Louise	storm 3	0.38	-2.488	0.020	-1.519	0.041
OLR-7	10/25/21	13:30	Rain	Louise	storm 3	0.38	-3.254	0.020	-7.390	0.019
OLR-8	10/25/21	14:00	Rain	Louise	storm 3	0.52	-3.664	0.031	-10.461	0.049
OLR-9	10/25/21	14:20	Rain	Louise	storm 3	0.42	-3.888	0.029	-13.350	0.073
OLR-10	10/25/21	14:35	Rain	Louise	storm 3	0.14	-4.051	0.037	-14.748	0.021

OLR-11	10/25/21	14:50	Rain	Louise	storm 3	0.26	-4.525	0.010	-18.303	0.171
OLR-12	10/25/21	15:05	Rain	Louise	storm 3	0.18	-4.553	0.005	-18.603	0.019
OLR-13	10/25/21	15:20	Rain	Louise	storm 3	0.08	-4.359	0.016	-22.531	0.025
OLR-14	10/25/21	15:35	Rain	Louise	storm 3	0.04	-4.797	0.027	-22.843	0.051
OLR-15	10/25/21	16:07	Rain	Louise	storm 3	0.06	-5.909	0.024	-35.118	0.073
OLR-16	10/25/21	16:30	Rain	Louise	storm 3	0.08	-6.344	0.029	-37.449	0.040
OLR-17	10/25/21	16:47	Rain	Louise	storm 3	0.02	-3.850	0.022	-22.426	0.043
OLS-4	10/25/21	15:03	Stream	Louise	storm 3		-4.100	0.040	-15.570	0.041
OLS-5	10/25/21	15:18	Stream	Louise	storm 3		-3.925	0.030	-14.816	0.141
OLS-7	10/25/21	16:15	Stream	Louise	storm 3		-3.869	0.032	-16.716	0.067
OHS-3	10/25/21	14:46	Stream	Henry	storm 3		-3.077	0.064	-10.203	0.075
OHS-4	10/25/21	15:01	Stream	Henry	storm 3		-2.894	0.024	-9.810	0.054
OHS-5	10/25/21	15:16	Stream	Henry	storm 3		-2.745	0.022	-9.368	0.015
OHS-6	10/25/21	15:31	Stream	Henry	storm 3		-3.338	0.025	-10.916	0.099
OHS-7	10/25/21	15:46	Stream	Henry	storm 3		-3.287	0.021	-11.053	0.140
OHS-8	10/25/21	16:01	Stream	Henry	storm 3		-3.328	0.013	-11.842	0.054
HIR-1	12/14/21	5:15	Integrated Rain	Rain Gauge	storm 4		-5.879	0.022	-19.655	0.037
HIR-2	12/14/21	9:15	Integrated Rain	Rain Gauge	storm 4		-6.803	0.042	-29.547	0.078
HIR-3	12/14/21	13:15	Integrated Rain	Rain Gauge	storm 4		-6.537	0.035	-28.354	0.023
DLR-1	12/14/21	5:15	Rain	Louise	storm 4	0.14	-7.158	0.028	-30.987	0.054
DLR-2	12/14/21	5:33	Rain	Louise	storm 4	0.28	-7.664	0.026	-36.228	0.046
DLR-3	12/14/21	5:48	Rain	Louise	storm 4	0.2	-7.059	0.037	-30.820	0.045
DLR-4	12/14/21	6:08	Rain	Louise	storm 4	0.36	-7.071	0.025	-30.611	0.024

DLR-5	12/14/21	6:28	Rain	Louise	storm 4	0.24	-7.784	0.008	-36.038	0.033
DLR-6	12/14/21	6:45	Rain	Louise	storm 4	0.2	-7.722	0.024	-35.609	0.046
DLR-7	12/14/21	7:05	Rain	Louise	storm 4	0.22	-7.390	0.018	-35.254	0.120
DLR-8	12/14/21	7:25	Rain	Louise	storm 4	0.4	-7.919	0.022	-38.453	0.079
DLR-9	12/14/21	7:45	Rain	Louise	storm 4	0.44	-7.713	0.018	-37.356	0.090
DLR-10	12/14/21	8:05	Rain	Louise	storm 4	0.4	-7.703	0.006	-38.647	0.078
DLR-11	12/14/21	8:25	Rain	Louise	storm 4	0.54	-7.327	0.010	-35.988	0.050
DLR-12	12/14/21	8:45	Rain	Louise	storm 4	0.48	-6.441	0.004	-29.115	0.061
DLR-13	12/14/21	9:05	Rain	Louise	storm 4	0.6	-5.779	0.037	-23.535	0.054
DLR-14	12/14/21	9:25	Rain	Louise	storm 4	0.44	-5.561	0.026	-22.737	0.055
DLR-15	12/14/21	9:45	Rain	Louise	storm 4	0.36	-5.455	0.020	-22.764	0.048
DLR-16	12/14/21	10:10	Rain	Louise	storm 4	0.3	-5.416	0.038	-22.445	0.055
DLR-17	12/14/21	10:30	Rain	Louise	storm 4	0.24	-5.704	0.008	-25.670	0.038
DLR-18	12/14/21	10:50	Rain	Louise	storm 4	0.26	-5.442	0.028	-26.768	0.009
DLR-19	12/14/21	11:10	Rain	Louise	storm 4	0.16	-4.820	0.038	-20.334	0.087
DLR-20	12/14/21	11:30	Rain	Louise	storm 4	0.34	-5.069	0.050	-20.863	0.038
DLR-21	12/14/21	11:55	Rain	Louise	storm 4	0.44	-4.863	0.026	-18.439	0.018
DLR-22	12/14/21	12:20	Rain	Louise	storm 4	0.16	-4.553	0.048	-17.018	0.061
DLR-23	12/14/21	12:40	Rain	Louise	storm 4	0.18	-4.206	0.008	-13.007	0.037
DLR-24	12/14/21	13:00	Rain	Louise	storm 4	0.24	-3.796	0.019	-15.589	0.107
DHR-1	12/14/21	6:00	Rain	Henry	storm 4	0.46	-7.083	0.062	-31.087	0.130
DHR-2	12/14/21	6:30	Rain	Henry	storm 4	0.4	-7.478	0.014	-34.264	0.067
DHR-3	12/14/21	7:00	Rain	Henry	storm 4	0.32	-7.749	0.008	-36.271	0.076

DHR-4	12/14/21	7:30	Rain	Henry	storm 4	0.6	-7.850	0.020	-37.808	0.065
DHR-5	12/14/21	8:00	Rain	Henry	storm 4	0.66	-7.850	0.007	-39.385	0.042
DHR-6	12/14/21	8:30	Rain	Henry	storm 4	0.74	-7.479	0.026	-36.943	0.109
DHR-7	12/14/21	9:00	Rain	Henry	storm 4	0.8	-6.471	0.055	-29.012	0.026
DHR-8	12/14/21	9:30	Rain	Henry	storm 4	0.72	-5.737	0.016	-23.727	0.137
DHR-9	12/14/21	10:00	Rain	Henry	storm 4	0.42	-5.632	0.046	-24.410	0.059
DHR-10	12/14/21	10:30	Rain	Henry	storm 4	0.38	-5.887	0.026	-26.477	0.087
DHR-11	12/14/21	10:40	Rain	Henry	storm 4	0.24	-6.436	0.003	-31.027	0.053
DHR-12	12/14/21	11:00	Rain	Henry	storm 4	0.08	-5.495	0.035	-27.548	0.110
DHR-13	12/14/21	11:30	Rain	Henry	storm 4	0.42	-4.384	0.023	-15.656	0.134
DHR-14	12/14/21	12:00	Rain	Henry	storm 4	0.3	-5.354	0.037	-22.147	0.071
DHR-15	12/14/21	12:30	Rain	Henry	storm 4	0.44	-5.186	0.026	-21.444	0.059
DHR-16	12/14/21	13:00	Rain	Henry	storm 4	0.32	-4.662	0.011	-18.539	0.043
DLS-1	12/14/21	5:00	Stream	Louise	storm 4		-6.294	0.071	-24.590	0.099
DLS-2	12/14/21	5:05	Stream	Louise	storm 4		-6.392	0.014	-25.358	0.154
DLS-3	12/14/21	5:15	Stream	Louise	storm 4		-6.451	0.050	-26.666	0.106
DLS-4	12/14/21	5:33	Stream	Louise	storm 4		-6.442	0.008	-27.616	0.071
DLS-5	12/14/21	5:48	Stream	Louise	storm 4		-6.440	0.020	-27.872	0.063
DLS-6	12/14/21	6:08	Stream	Louise	storm 4		-6.561	0.023	-28.065	0.035
DLS-7	12/14/21	6:28	Stream	Louise	storm 4		-7.142	0.037	-32.011	0.092
DLS-8	12/14/21	6:45	Stream	Louise	storm 4		-7.240	0.031	-32.846	0.020
DLS-9	12/14/21	7:05	Stream	Louise	storm 4		-7.221	0.012	-33.169	0.071
DLS-10	12/14/21	7:25	Stream	Louise	storm 4		-7.454	0.011	-35.501	0.050

DLS-11	12/14/21	7:45	Stream	Louise	storm 4		-7.447	0.017	-35.974	0.024
DLS-12	12/14/21	8:05	Stream	Louise	storm 4		-7.621	0.015	-37.584	0.055
DLS-13	12/14/21	8:25	Stream	Louise	storm 4		-7.574	0.008	-36.934	0.085
DLS-14	12/14/21	8:45	Stream	Louise	storm 4		-7.153	0.040	-33.744	0.108
DLS-15	12/14/21	9:05	Stream	Louise	storm 4		-6.760	0.017	-31.271	0.089
DLS-16	12/14/21	9:25	Stream	Louise	storm 4		-6.495	0.036	-29.974	0.156
DLS-17	12/14/21	9:45	Stream	Louise	storm 4		-6.451	0.043	-29.093	0.049
DLS-18	12/14/21	10:10	Stream	Louise	storm 4		-6.401	0.019	-29.362	0.142
DLS-19	12/14/21	10:30	Stream	Louise	storm 4		-5.952	0.026	-27.154	0.047
DLS-20	12/14/21	10:37	Stream	Louise	storm 4		-6.371	0.029	-29.859	0.144
DLS-21	12/14/21	10:50	Stream	Louise	storm 4		-6.371	0.012	-30.060	0.099
DLS-22	12/14/21	11:10	Stream	Louise	storm 4		-6.424	0.097	-29.491	0.214
DLS-23	12/14/21	11:30	Stream	Louise	storm 4		-6.197	0.011	-28.708	0.167
DLS-24	12/14/21	11:55	Stream	Louise	storm 4		-6.000	0.037	-27.265	0.122
DLS-25	12/14/21	12:20	Stream	Louise	storm 4		-5.960	0.034	-26.945	0.085
DLS-26	12/14/21	12:40	Stream	Louise	storm 4		-6.009	0.026	-27.335	0.070
DLS-27	12/14/21	13:00	Stream	Louise	storm 4		-6.102	0.033	-28.520	0.049
DLS-28	12/14/21	13:20	Stream	Louise	storm 4		-6.284	0.040	-29.449	0.159
DHS-1	12/14/21	5:30	Stream	Henry	storm 4		-5.706	0.029	-25.153	0.210
DHS-2	12/14/21	6:00	Stream	Henry	storm 4		-5.687	0.009	-24.862	0.045
DHS-3	12/14/21	6:30	Stream	Henry	storm 4		-6.664	0.010	-30.609	0.028
DHS-4	12/14/21	7:00	Stream	Henry	storm 4		-7.121	0.036	-33.140	0.014
DHS-5	12/14/21	7:30	Stream	Henry	storm 4		-7.275	0.038	-34.660	0.116

DHS-6	12/14/21	8:00	Stream	Henry	storm 4		-7.590	0.039	-37.188	0.106
DHS-7	12/14/21	8:30	Stream	Henry	storm 4		-7.235	0.020	-35.205	0.064
DHS-8	12/14/21	9:00	Stream	Henry	storm 4		-6.857	0.040	-32.181	0.053
DHS-9	12/14/21	9:30	Stream	Henry	storm 4		-6.536	0.038	-29.917	0.018
DHS-10	12/14/21	10:00	Stream	Henry	storm 4		-6.406	0.054	-29.165	0.076
DHS-11	12/14/21	10:30	Stream	Henry	storm 4		-6.253	0.030	-28.707	0.152
DHS-12	12/14/21	10:40	Stream	Henry	storm 4		-6.267	0.034	-29.707	0.047
DHS-13	12/14/21	11:00	Stream	Henry	storm 4		-6.333	0.020	-29.923	0.069
DHS-14	12/14/21	11:30	Stream	Henry	storm 4		-6.321	0.045	-29.181	0.128
DHS-15	12/14/21	12:00	Stream	Henry	storm 4		-5.910	0.044	-25.980	0.065
DHS-16	12/14/21	12:30	Stream	Henry	storm 4		-6.122	0.053	-28.047	0.169
DHS-17	12/14/21	13:00	Stream	Henry	storm 4		-6.140	0.005	-28.144	0.023
GIR-3	12/23/21	11:00	Integrated Rain	Rain Gauge	storm 5		-7.104	0.025	-43.511	0.074
GIR-4	12/23/21	14:00	Integrated Rain	Rain Gauge	storm 5		-8.536	0.049	-54.625	0.043
DLR-50	12/23/21	11:38	Rain	Louise	storm 5	0.06	-11.222	0.037	-72.709	0.109
DLR-51	12/23/21	12:00	Rain	Louise	storm 5	0.08	-11.199	0.012	-71.516	0.077
DLR-52	12/23/21	12:18	Rain	Louise	storm 5	0.2	-11.222	0.034	-72.028	0.041
DLR-53	12/23/21	12:37	Rain	Louise	storm 5	0.1	-10.796	0.025	-69.205	0.112
DLR-54	12/23/21	12:58	Rain	Louise	storm 5	0.18	-11.133	0.008	-70.946	0.071
DLR-55	12/23/21	13:16	Rain	Louise	storm 5	0.18	-10.941	0.062	-69.448	0.444
DLR-56	12/23/21	13:35	Rain	Louise	storm 5	0.22	-11.030	0.023	-68.786	0.121
DLR-57	12/23/21	13:53	Rain	Louise	storm 5	0.24	-11.447	0.013	-72.844	0.058
DHR-50	12/23/21	12:05	Rain	Henry	storm 5	0.16	-10.144	0.017	-66.774	0.104

DHR-51	12/23/21	12:27	Rain	Henry	storm 5	0.14	-9.869	0.013	-64.524	0.027
DHR-52	12/23/21	12:47	Rain	Henry	storm 5	0.18	-9.895	0.019	-64.711	0.108
DHR-53	12/23/21	13:07	Rain	Henry	storm 5	0.18	-9.697	0.033	-62.036	0.011
DHR-54	12/23/21	13:25	Rain	Henry	storm 5	0.16	-9.884	0.037	-63.261	0.100
DHR-55	12/23/21	13:45	Rain	Henry	storm 5	0.3	-10.155	0.022	-64.738	0.020
DLS-50	12/23/21	11:15	Stream	Louise	storm 5		-10.376	0.025	-67.398	0.076
DLS-51	12/23/21	11:37	Stream	Louise	storm 5		-10.009	0.013	-64.177	0.039
DLS-52	12/23/21	11:55	Stream	Louise	storm 5		-10.148	0.046	-64.109	0.027
DLS-53	12/23/21	12:16	Stream	Louise	storm 5		-10.193	0.063	-64.959	0.048
DLS-54	12/23/21	12:35	Stream	Louise	storm 5		-10.220	0.010	-65.394	0.071
DLS-55	12/23/21	12:56	Stream	Louise	storm 5		-10.555	0.024	-66.392	0.044
DLS-56	12/23/21	13:15	Stream	Louise	storm 5		-10.271	0.030	-64.048	0.118
DLS-57	12/23/21	13:34	Stream	Louise	storm 5		-10.056	0.003	-62.413	0.044
DLS-58	12/23/21	13:53	Stream	Louise	storm 5		-10.248	0.027	-63.590	0.074
DHS-50	12/23/21	11:25	Stream	Henry	storm 5		-9.936	0.017	-65.111	0.136
DHS-51	12/23/21	11:46	Stream	Henry	storm 5		-10.204	0.003	-65.440	0.049
DHS-52	12/23/21	12:05	Stream	Henry	storm 5		-9.986	0.033	-64.341	0.061
DHS-53	12/23/21	12:26	Stream	Henry	storm 5		-10.261	0.028	-64.261	0.025
DHS-54	12/23/21	12:47	Stream	Henry	storm 5		-10.232	0.017	-64.231	0.084
DHS-55	12/23/21	13:06	Stream	Henry	storm 5		-9.949	0.049	-61.371	0.067
DHS-56	12/23/21	13:24	Stream	Henry	storm 5		-9.762	0.014	-59.930	0.043
DHS-57	12/23/21	13:45	Stream	Henry	storm 5		-9.900	0.009	-60.386	0.058
GIR-5	12/24/21	6:01	Integrated Rain	Rain Gauge	storm 5		-9.703	0.021	-63.217	0.066

DGR-50	12/24/21	7:11	Rain	Rain Gauge	storm 5	0.04	-7.106	0.029	-36.127	0.121
DGR-51	12/24/21	7:30	Rain	Rain Gauge	storm 5		-6.802	0.033	-37.280	0.079
DLS-59	12/24/21	5:35	Stream	Louise	storm 5		-8.553	0.020	-49.473	0.121
DLS-60	12/24/21	6:05	Stream	Louise	storm 5		-8.331	0.018	-48.511	0.057
DLS-61	12/24/21	6:31	Stream	Louise	storm 5		-8.422	0.015	-48.754	0.111
DLS-62	12/24/21	6:50	Stream	Louise	storm 5		-8.417	0.030	-48.225	0.052
DLS-63	12/24/21	7:13	Stream	Louise	storm 5		-8.212	0.027	-46.862	0.090
DLS-64	12/24/21	7:35	Stream	Louise	storm 5		-8.186	0.012	-46.605	0.076
DLS-65	12/24/21	7:54	Stream	Louise	storm 5		-8.450	0.027	-48.246	0.104
DLS-66	12/24/21	8:15	Stream	Louise	storm 5		-8.384	0.019	-48.300	0.146
DHS-58	12/24/21	5:47	Stream	Henry	storm 5		-8.770	0.018	-50.866	0.064
DHS-59	12/24/21	6:20	Stream	Henry	storm 5		-8.787	0.028	-50.603	0.128
DHS-60	12/24/21	6:41	Stream	Henry	storm 5		-8.609	0.017	-49.988	0.066
DHS-61	12/24/21	7:03	Stream	Henry	storm 5		-8.455	0.037	-48.766	0.082
DHS-62	12/24/21	7:23	Stream	Henry	storm 5		-8.195	0.022	-45.704	0.140
DHS-63	12/24/21	7:45	Stream	Henry	storm 5		-8.462	0.001	-47.894	0.037
DHS-64	12/24/21	8:03	Stream	Henry	storm 5		-8.643	0.039	-49.394	0.107
GIR-6	12/28/21	13:00	Integrated Rain	Rain Gauge			-5.885	0.016	-24.134	0.096
DLS-67	12/28/21	13:00	Stream	Louise			-7.938	0.037	-44.779	0.009
GIR-7	1/4/22	13:00	Integrated Rain	Rain Gauge	storm 6		-8.364	0.030	-45.304	0.061
JLS-1	1/4/22	13:00	Stream	Louise	storm 6		-8.162	0.027	-47.096	0.126
MLR-3	3/28/22	10:00	Rain	Louise	storm 7	0.02	-3.720	0.026	-13.630	0.100

MLR-4	3/28/22	10:30	Rain	Louise	storm 7	0.12	-4.228	0.054	-14.741	0.137
MLR-5	3/28/22	11:00	Rain	Louise	storm 7	0.14	-5.306	0.039	-21.715	0.058
MLR-6	3/28/22	11:30	Rain	Louise	storm 7	0.22	-6.196	0.037	-27.930	0.070
MLR-7	3/28/22	12:00	Rain	Louise	storm 7	0.26	-6.141	0.021	-26.623	0.046
MLR-8	3/28/22	12:30	Rain	Louise	storm 7	0.18	-5.861	0.013	-24.438	0.024
MLR-9	3/28/22	13:00	Rain	Louise	storm 7	0.5	-7.111	0.023	-35.197	0.063
MLR-10	3/28/22	13:30	Rain	Louise	storm 7	0.44	-7.794	0.097	-41.819	0.883
MLR-11	3/28/22	14:00	Rain	Louise	storm 7	0.22	-7.541	0.020	-43.250	0.027
MLR-12	3/28/22	14:30	Rain	Louise	storm 7	0.02	-7.137	0.022	-40.147	0.114
MHR-1	3/28/22	10:05	Rain	Henry	storm 7	0.02	-6.809	0.037	-38.843	0.065
MHR-2	3/28/22	10:30	Rain	Henry	storm 7	0.12	-4.006	0.014	-14.678	0.106
MHR-3	3/28/22	11:00	Rain	Henry	storm 7	0.14	-4.453	0.014	-15.445	0.237
MLS-1	3/28/22	12:30	Stream	Louise	storm 7		-5.871	0.044	-25.514	0.096
MLS-2	3/28/22	13:00	Stream	Louise	storm 7		-6.865	0.036	-33.324	0.015
MLS-3	3/28/22	13:30	Stream	Louise	storm 7		-7.281	0.033	-37.870	0.066
MLS-4	3/28/22	14:00	Stream	Louise	storm 7		-7.269	0.033	-38.742	0.006
MLS-5	3/28/22	14:30	Stream	Louise	storm 7		-7.117	0.028	-38.263	0.045
MLS-6	3/28/22	15:00	Stream	Louise	storm 7		-7.131	0.010	-37.558	0.103
MHS-1	3/28/22	12:40	Stream	Henry	storm 7		-6.081	0.042	-26.931	0.053
MHS-2	3/28/22	13:03	Stream	Henry	storm 7		-6.244	0.024	-30.633	0.068
MHS-3	3/28/22	13:33	Stream	Henry	storm 7		-6.893	0.030	-34.514	0.099
MHS-4	3/28/22	14:03	Stream	Henry	storm 7		-6.961	0.035	-34.666	0.005
MHS-5	3/28/22	14:33	Stream	Henry	storm 7		-6.949	0.032	-34.645	0.146

MHS-6	3/28/22	15:00	Stream	Henry	storm 7	-6.987	0.024	-34.781	0.048
MGIR-1	3/28/22	16:00	Integrated Rain	Rain Gauge	storm 7	-6.578	0.007	-32.191	0.070
MRIR-1	3/28/22	16:00	Integrated Rain	Ridge	storm 7	-6.931	0.041	-34.310	0.053
MHIR-1	3/28/22	16:00	Integrated Rain		storm 7	-6.744	0.020	-32.997	0.004

Table S5. Resistivity survey and model metrics at Henry, Louise, and Thelma. "RMS" shows root mean square misfit.

**Survey date**	**Contact resistance range (kohms)**	**% data used after filtering**	**R-squared value of reciprocal error model**	**Inversion RMS misfit**
Henry				
December 12, 2020	8 to 58	97.08	0.891	1.71
February 2, 2021	5.57 to 13.49	96.35	0.952	1.03
February 9, 2021	5.54 to 26.79	96.29	0.873	0.98
February 16, 2021	3.96 to 13.93	96.53	0.934	1
February 23, 2021	4.79 to 14.07	96.71	0.927	1.34
March 1, 2021	4.94 to 16.55	96.35	0.938	1
March 12, 2021	2.98 to 12.2	96.41	0.942	1.77
April 24, 2021	8.99 to 67.27	96.47	0.885	1.2
June 4, 2021	5 to 50	96.71	0.767	1.16
October 23, 2021	103.72 to 421.63	96.11	0.838	1.06
October 26, 2021	-	93.16	0.970	1.17
December 21, 2021	3 to 19	96.02	0.952	1.44
January 4, 2022	4 to 15	95.63	0.851	1.49
March 11, 2022	4.76 to 30.51	95.92	0.895	1.05
May 18, 2022	5.62 to 49.73	96.17	0.871	1.01
Louise				
December 11, 2020	13 to 60	93.43	0.887	1.18

February 2, 2021	2.57 to 16.41	94.65	0.874	0.86
February 9, 2021	4.48 to 21.75	95.21	0.921	1.14
February 16, 2021	5.54 to 27.19	95.78	0.895	1
February 23, 2021	4.52 to 15.9	95.21	0.915	1.1
March 2, 2021	3.67 to 17.07	95.13	0.903	1.97
March 12, 2021	2 to 16	93.92	0.877	1.11
April 24, 2021	2 to 24.5	94.73	0.932	1.01
June 2, 2021	2 to 50	95.13	0.779	1
October 23, 2021	20 to 300	93.23	0.806	2.15
October 26, 2021	2.28 to 17.8	96.67	0.970	1.17
December 21, 2021	1.18 to 12	95.54	0.887	1.29
January 4, 2022	1.33 to 12	96.67	0.893	1.50
March 11, 2022	1.59 to 16.46	95.65	0.891	1.35
May 18, 2022	2.12 to 59.58	95.82	0.863	1.01
Thelma				
December 13, 2020	10 to 60	96.65	0.877	1.33
February 1, 2021	3.43 to 20.36	97.26	0.972	1.00
February 9, 2021	5.39 to 21.21	95.44	0.924	1.06
February 16, 2021	4.36 to 17.34	97.32	0.946	1.00
February 23, 2021	5.98 to 17.12	97.26	0.961	1.04
March 2, 2021	6.63 to 18.65	96.04	0.956	1.03
March 12, 2021	2.5 to 14.3	96.17	0.971	1.00
April 24, 2021	5.8 to 18.44	96.84	0.924	1.00
June 5, 2021	5 to 50	97.14	0.805	1.09
October 26, 2021	4 to 14	97.62	0.884	1.40
March 11, 2022	5.93 to 22.59	97.14	0.845	1.22

Supplementary Information References

1. PRISM Climate Group, O. S. U. https://prism.oregonstate.edu (2014).
2. Septentrio Satellite Navigation. Septentrio Reference Guide. https://www.septentrio.com/rx_refguides/ssrc4/refguide.html (2016).
3. Dansgaard, W. Stable isotopes in precipitation. *Tellus* **16**, 436–468 (1964).
4. Rozanski, K., Araguás-Araguás, L. & Gonfiantini, R. Isotopic Patterns in Modern Global Precipitation. 1–36 (1993) doi:10.1029/gm078p0001.
5. Whalley, W. R. *et al.* Methods to estimate changes in soil water for phenotyping root activity in the field. *Plant and Soil* **415**, 407–422 (2017).
6. Kotikian, M., Parsekian, A. D., Paige, G. & Carey, A. Observing Heterogeneous Unsaturated Flow at the Hillslope Scale Using Time-Lapse Electrical Resistivity Tomography. *Vadose Zone Journal* **18**, 1–16 (2019).
7. Blanchy, G., Saneiyan, S., Boyd, J., McLachlan, P. & Binley, A. ResIPy, an intuitive open source software for complex geoelectrical inversion/modeling. *Computers & Geosciences* **137**, 104423 (2020).
8. Slater, L., Binley, A. M., Daily, W. & Johnson, R. Cross-hole electrical imaging of a controlled saline tracer injection. *Journal of Applied Geophysics* **44**, (2000).
9. Koestel, J., Kemna, A., Javaux, M., Binley, A. & Vereecken, H. Quantitative imaging of solute transport in an unsaturated and undisturbed soil monolith with 3-D ERT and TDR. *Water Resources Research* **44**, (2008).
10. Binley, A. & Kemna, A. DC resistivity and induced polarization methods. *Hydrogeophysics* 129–156 (2005).
11. LaBrecque, D. J. & Yang, X. Difference inversion of ERT data: A fast inversion method for 3-D in situ monitoring. *Journal of Environmental & Engineering Geophysics* **6**, 83–89 (2001).
12. Oldenburg, D. W. & Li, Y. Estimating depth of investigation in dc resistivity and IP surveys. *Geophysics* **64**, 403–416 (1999).

13. Nijland, W., Meijde, M. van der, Addink, E. A. & Jong, S. M. de. Detection of soil moisture and vegetation water abstraction in a mediterranean natural area using electrical resistivity tomography. *Catena* **81**, 209–216 (2010).
14. Binley, A. & Slater, L. *Resistivity and induced polarization: Theory and applications to the near-surface earth*. (Cambridge University Press, 2020).
15. Carey, A. M., Paige, G. B., Carr, B. J. & Dogan, M. Forward modeling to investigate inversion artifacts resulting from time-lapse electrical resistivity tomography during rainfall simulations. *Journal of Applied Geophysics* **145**, 39–49 (2017).
16. Hayley, K., Bentley, L. R., Gharibi, M. & Nightingale, M. Low temperature dependence of electrical resistivity: Implications for near surface geophysical monitoring. *Geophysical Research Letters* **34**, (2007).
17. Brunet, P., Clément, R. & Bouvier, C. Monitoring soil water content and deficit using Electrical Resistivity Tomography (ERT) - A case study in the Cevennes area, France. *Journal of Hydrology* **380**, 146–153 (2010).
18. Michot, D. *et al.* Spatial and temporal monitoring of soil water content with an irrigated corn crop cover using surface electrical resistivity tomography. *Water Resources Research* **39**, (2003).
19. Yeh, T. C. J. *et al.* A geostatistically based inverse model for electrical resistivity surveys and its applications to vadose zone hydrology. *Water Resources Research* **38**, 14-1-14–13 (2002).
20. Fisher, J. B. *et al.* ECOSTRESS: NASA's Next Generation Mission to Measure Evapotranspiration From the International Space Station. *Water Resources Research* **56**, (2020).
21. Wilder, B. A. & Kinoshita, A. M. Incorporating ECOSTRESS evapotranspiration in a paired catchment water balance analysis after the 2018 Holy Fire in California. *Catena* **215**, (2022).
22. McGuire, L. A., Rengers, F. K., Kean, J. W., Staley, D. M. & Mirus, B. B. Incorporating spatially heterogeneous infiltration capacity into hydrologic models with applications for simulating post-wildfire debris flow initiation. *Hydrological Processes* **32**, 1173–1187 (2018).
23. Moody, J. A., Martin, R. G. & Ebel, B. A. Sources of inherent infiltration variability in postwildfire soils. *Hydrological Processes* **33**, 3010–3029 (2019).

24. Pierson, F. B., Robichaud, P. R. & Spaeth, K. E. Spatial and temporal effects of wildfire on the hydrology of a steep rangeland watershed. *Hydrological Processes* **15**, 2905–2916 (2001).

25. Stoof, C. R. *et al.* Hydrological response of a small catchment burned by experimental fire. *Hydrology and Earth System Sciences* **16**, 267–285 (2012).

26. Wall, S. A., Roering, J. J. & Rengers, F. K. Runoff-initiated post-fire debris flow Western Cascades, Oregon. *Landslides* **17**, 1649–1661 (2020).

27. Ritsema, C. J. & Dekker, L. W. Distribution Flow: A General Process in the Top Layer of Water Repellent Soils. *Water Resources Research* **31**, 1187–1200 (1995).

28. Krammes, J. S. & Debano, L. F. Soil wettability: a neglected factor in watershed management. *Water Resources Research* **1**, 283–286 (1965).

29. Onda, Y., Dietrich, W. E. & Booker, F. Evolution of overland flow after a severe forest fire, Point Reyes, California. *Catena* **72**, (2008).
